# Adverse Effects of Anastomotic Leakage on Local Recurrence and Survival After Curative Anterior Resection for Rectal Cancer: A Systematic Review and Meta-analysis

**DOI:** 10.1007/s00268-016-3761-1

**Published:** 2016-10-14

**Authors:** Shuanhu Wang, Jingjing Liu, Shan Wang, Hongyun Zhao, Sitang Ge, Wenbin Wang

**Affiliations:** 1 Department of Gastrointestinal Surgery, The First Affiliated Hospital of Bengbu Medical College, Bengbu, Anhui China; 2 Department of General Surgery, The Forth Affiliated Hospital of Anhui Medical University, Hefei, Anhui China

## Abstract

**Background:**

Anastomotic leakage is a serious complication associated with anterior resection for rectal cancer, the long-term effects of which are unclear. Therefore, a systematic review and meta-analysis were conducted to evaluate the impact of anastomotic leakage on disease recurrence and survival.

**Methods:**

We searched PubMed, Embase, and the Cochrane Library databases from their inception to January 2016. Studies evaluating the oncologic impact of anastomotic leakage were included in the meta-analysis. Outcome measures were local recurrence, overall survival, cancer-specific survival, and distant recurrence. Pooled hazard ratio (HR) with 95 % confidence interval (CI) was calculated using random effects models.

**Results:**

Fourteen studies containing 11,353 patients met inclusion criteria. Anastomotic leakage was associated with a greater local recurrence (HR 1.71; 95 % CI 1.22–2.38) and decreased in both overall survival (HR 1.67; 95 % CI 1.19–2.35) and cancer-specific survival (HR 1.30; 95 % CI 1.08–1.56); anastomotic leakage did not increase distant recurrence (HR 1.03; 95 % CI 0.76–1.40).

**Conclusions:**

Anastomotic leakage was associated with high local recurrence and poor survival (both overall and cancer-specific), but not with distant recurrence.

## Introduction

Because of advances in operative techniques and our knowledge of the biology of rectal cancer, an increasing number of patients with rectal cancer have undergone sphincter preserving surgery. Anastomotic leakage, however, is a serious surgical complication with an incidence that varies from 4 to 29.5 % [1, 2] and is associated with short-term mortality, high reoperation rate, and increased healthcare costs [3–5].

The long-term outcome of curative rectal cancer resections is affected by many factors, such as lower differentiation, later stage, and older age [6, 7]. Though some studies found that anastomotic leakage was associated with a poorer long-term outcome [8, 9], others did not [10, 11]. Thus, we performed a systematic review and meta-analysis to determine the evidence-based impact of anastomotic leakage on long-term outcomes after curative anterior resection.

## Materials and methods

### Literature search and inclusion criteria

Two authors (S.W. and J.L.) searched independently the electronic databases (inception to January 2016) of Pubmed, Embase, and the Cochrane Library. Search terms included the following keywords in various combinations: “rectal neoplasms,” “anastomotic leak,” “recurrence,” “neoplasm metastasis,” “survival,” and “mortality.” Searches of subject headings (MeSH) and text words were performed with no language restrictions applied. We followed the Cochrane approach of PICO (population intervention, comparison, outcome, and context). We also searched for references included in the articles to identify related studies.

For this meta-analysis, we followed the PRISMA guidelines [12]. The inclusion criteria were as follows: (1) patients who underwent a curative anterior resection for rectal cancer; (2) studies that analyzed the impact of anastomotic leakage on long-term outcomes, including local recurrence, overall survival, cancer-specific survival, or distant recurrence; and (3) patients with and without anastomotic leakage who were compared using a multivariate Cox proportional hazards model.

Conversely, the exclusion criteria were as follows: (1) patients who underwent an emergency operation; and (2) studies including all anatomic locations of colorectal cancer, unless the data were presented separately.

Anastomotic leakage was defined as a communication between the intra- and extraluminal compartments, determined by either clinical or radiologic evidence [13]. Local recurrence was defined as a mass in the lesser pelvis documented by clinical, radiologic, or pathologic examination. Distant recurrence was defined as tumor growth in any lymph node outside the pelvis, or in any other organ documented by clinical, radiologic, or pathologic examination.

### Data extraction, outcome measures, and quality assessment

Disagreements regarding the independently extracted data (by authors S.W. and S.G.) were settled through discussion, when no consensus could be reached, a third specialist was consulted (W.W.). For this study, outcome measures evaluated included local recurrence, overall survival, cancer-specific survival, and distant recurrence.

The quality of the included studies was assessed by two independent authors (S.W. and H.Z.) using the criteria shown in File S1. The guideline for appraising the studies was adopted from a quality assessment framework for systematic reviews of prognostic studies [14]. This framework includes the following six areas of potential bias: study participation, study attrition, measurement of prognostic factors, measurement of and controlling for confounding variables, measurement of outcomes, and approaches in analysis.

### Statistical analysis

Pooled HR and 95 % CI were estimated for each outcome. Statistical heterogeneity was assessed with *I* ^2^ and χ^2^ statistics. Heterogeneity was considered significant if the *p* value (χ^2^) was <0.1 and *I* ^2^ was >50 %. A random effects model was used regardless of heterogeneity [15]. Whenever significant heterogeneity was present, potential sources of heterogeneity were assessed. For example, a sensitivity analysis was performed, and the study was excluded if the results were outside the range established by others. Potential publication bias was assessed through visual inspection of Begg’s funnel plots where the log HR was plotted against their standard errors. The presence of publication bias was then evaluated using the Begg’s test [16]. Statistical analysis was performed using Stata 12.0 (Stata Corporation, College Station, TX, USA) and RevMan 5.3 (Nordic Cochrane Centre, Cochrane Collaboration, Copenhagen, Denmark).

## Results

### Search results and study descriptions

**Fig. 1:**
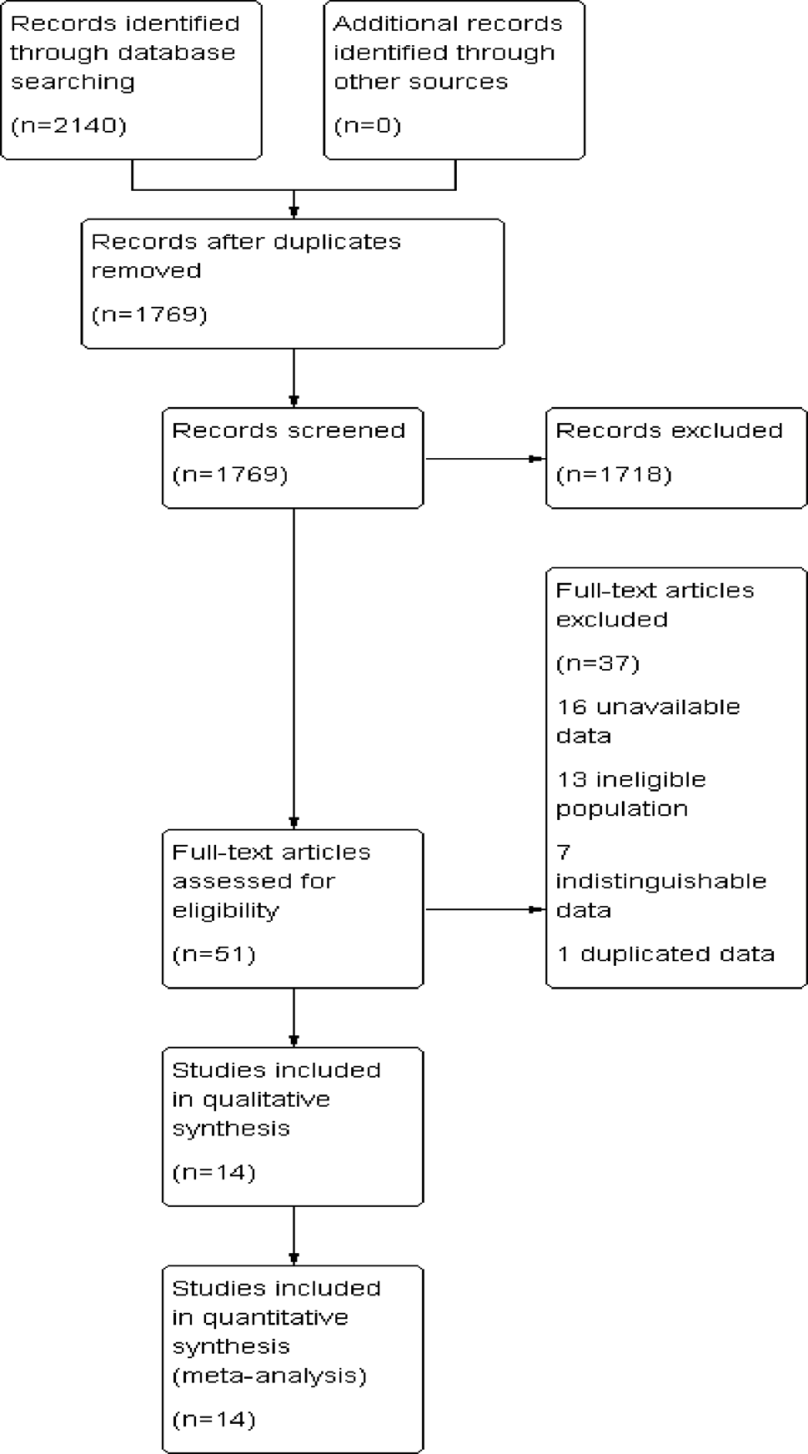
Prisma flow diagram of the literature screening and selection

The predefined search strategy identified 2140 studies, and after removal of 371 duplicate studies, 1769 articles remained. A total of 1718 studies were excluded after reading the titles and abstracts, mainly because they were not pertinent to the topic, leaving 51 studies for full-text review. After further review, 37 studies were excluded for the following reasons: 16 studies had unavailable data, 10 studies included patients who underwent palliative operations, 7 studies included colorectal cancer with data unable to be sorted specifically to anterior resection for rectal cancer, 3 studies included patients who underwent emergency operations, and 1 study contained duplicate data. Finally, 14 studies were included in this meta-analysis [17–30] as seen in the PRISMA flow diagram (Fig. 1).

All included studies were published between 2001 and 2015. Sample size ranged from 108 to 2480 patients, giving a total of 11,353 patients available for inclusion. Seven studies were prospective cohort studies, and seven were retrospective cohort studies. Furthermore, nine studies provided data on local recurrence, ten provided data on overall survival, seven examined cancer-specific survival, and three assessed distant recurrence. The multivariate Cox proportional hazards model was applied to all 14 studies to adjust for potential confounding data. Further characteristics of these studies are presented in Table 1.

**Table 1:** Characteristics of included articles

References	Year	Country	Period	Journal	Sample size	Definition of AL	Follow-up in years^a^
Bell et al. [17]	2003	Australia	1971–1991	Br J Surg	403	Clinical or radiological	10.80 (5–23)
Bertelsen et al. [18]	2010	Denmark	2001–2004	Colorectal Dis	1494	Clinical	3.80 (0.09–6.18)
den Dulk et al. [19]	2009	Multination	1987–2003	Br J Surg	2480	Clinical	5.90 (0.20–14.90)
Ebinger et al. [20]	2015	Switzerland	1991–2010	Int J Colorectal Dis	584	Clinical or radiological	5.20 (0.20–21.20)
Espin et al. [21]	2015	Spain	2006–2008	Br J Surg	1153	Clinical	At least 5
Gong et al. [22]	2014	China	2003–2007	Asian Pac J Cancer Prev	460	Clinical	3.50
Gunkova et al. [23]	2013	Czech	2001–2009	Rozhl Chir	174	Clinical or radiological	NA
Jager et al. [24]	2015	Osterreich	2003–2010	Chirurg	108	Clinical or radiological	5.80 (2–10.30)
Jorgren et al. [25]	2011	Sweden	1995–1997	Colorectal Dis	250	Clinical	5
Kang et al. [26]	2015	Korea	2006–2009	Medicine (Baltimore)	1083	Clinical	4.50 (0.08–7.70)
Ke et al. [27]	2015	China	2007–2011	Zhonghua Wei Chang Wai Ke Za Zhi	653	Clinical or radiological	3.90 (0.10–7.58)
Kulu et al. [28]	2015	Multination	2002–2011	Ann Surg Oncol	570	Clinical or radiological	4.70 ± 2.90
Merkel et al. [29]	2001	Germany	1978–1996	Colorectal Dis	814	Clinical	7.50 (0.30–20.80)
Smith et al. [30]	2012	USA	1991–2010	Ann Surg	1127	Clinical	6.20 (IQR 1.60–8.90)

*NA* not available, *AL* anastomotic leakage, *IQR* interquartile range

^a^Median (range)

### Study quality

We used 14 quality domains reflecting 6 main quality aspects in the framework established by Hayden et al. [14]. Each domain was evaluated using a quality score of 0, 0.5, or 1. The total score was obtained through the addition of each domain, thereby making the maximum quality score 14. The median (interquartile range) of the total quality score was 12 (10.6, 12.5). The results of the quality assessment of the included studies are shown in Table 2.

**Table 2:** Quality assessment of included articles

References	Study participation max 3 pts	Study attrition max 2 pts	Prognostic factor measurement max 2 pts	Outcome measurement max 2 pts	Confounding measurement and account max 2 pts	Analysis max 3 pts	Total score max 14 pts
Bell et al. [17]	3	2	2	2	1.5	2.5	13
Bertelsen et al. [18]	3	2	2	1	1.5	3	12.5
den Dulk et al. [19]	3	2	2	2	1	2.5	12.5
Ebinger et al. [20]	3	2	2	2	1.5	3	13.5
Espin et al. [21]	3	1	2	2	1	3	12
Gong et al. [22]	3	2	2	2	1	2	12
Gunkova et al. [23]	NA	NA	NA	NA	NA	NA	NA
Jager et al. [24]	NA	NA	NA	NA	NA	NA	NA
Jorgren et al. [25]	3	1	2	2	1	2	11
Kang et al. [26]	1.5	1	2	2	1	3	10.5
Ke et al. [27]	1.5	1	1	1	1	1.5	7
Kulu et al. [28]	3	2	2	1.5	1	3	12.5
Merkel et al. [29]	3	1	2	2	1	2.5	11.5
Smith et al. [30]	1.5	2	2	1	1	3	10.5

*Max* maximum, *pts* points, *NA* not available

### Anastomotic leakage and local recurrence

**Fig. 2:**
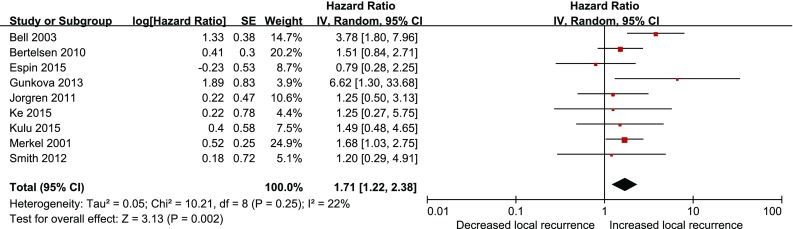
Effect of anastomotic leakage on the risk of local recurrence of rectal cancer after anterior resection

Nine studies reported local recurrence after anastomotic leakage, with no significant heterogeneity among them (*P* = 0.25, *I* ^2^ = 22 %). In the random effects model, anastomotic leakage was significantly associated with greater local recurrence rate (HR 1.71; 95 % CI 1.22–2.38; *P* = 0.002), as shown in Fig. 2.

### Anastomotic leakage and overall survival

**Fig. 3:**
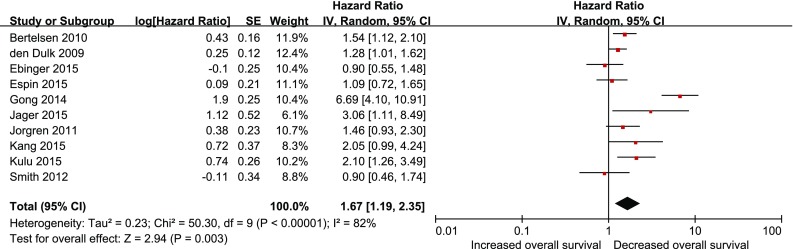
Effect of anastomotic leakage on overall survival

Ten studies assessed overall survival after anastomotic leakage. Our meta-analysis found that anastomotic leakage decreased overall survival (HR 1.67; 95 % CI 1.19–2.35; *P* = 0.003, Fig. 3), but with significant heterogeneity among studies (*P* < 0.00001, *I* ^2^ = 82 %). As shown in Fig. 3, the results of the study conducted by Gong et al. were notably outside of the range established by others, probably contributing to this heterogeneity. After excluding this study, results indicated that anastomotic leakage was associated with poor overall survival (HR 1.38; 95 % CI 1.14–1.66; *P* = 0.001) and no significant heterogeneity was observed among the remaining studies (*P* = 0.13, *I* ^2^ = 37 %).

### Anastomotic leakage and cancer-specific survival

**Fig. 4:**
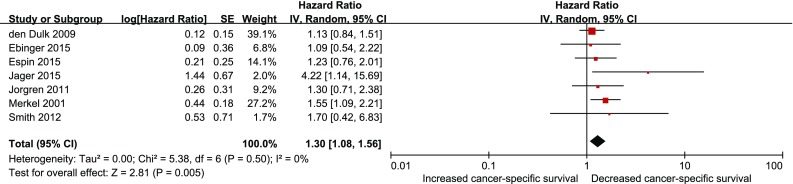
Effect of anastomotic leakage on cancer-specific survival

In the random effects model, anastomotic leakage was associated with lesser cancer-specific survival (HR 1.30; 95 % CI 1.08–1.56; *P* = 0.005), as shown in Fig. 4. There was no heterogeneity among the seven studies that assessed cancer-specific survival after anastomotic leakage (*P* = 0.50, *I* ^2^ = 0 %).

### Anastomotic leakage and distant recurrence

**Fig. 5:**
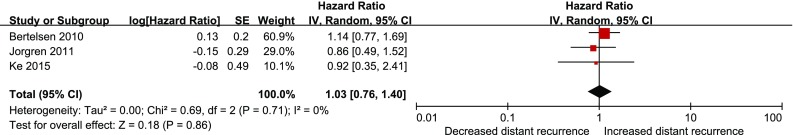
Effect of anastomotic leakage on distant recurrence

Three studies provided data on distant recurrence after anastomotic leakage. Results showed that anastomotic leakage had no significant effect on distant recurrence (1.03; 95 % CI 0.76–1.40, *P* = 0.86, Fig. 5), and there was no heterogeneity among the studies (*P* = 0.71, *I* ^2^ = 0 %).

### Publication bias

Assessment of publication bias revealed no potential publication bias among the included studies (Begg’s test, *P* = 0.18).

## Discussion

Our study evaluated the oncologic impact of anastomotic leakage after a “curative” anterior resection for rectal cancer. The results of the present meta-analysis revealed that anastomotic leakage was associated with worse overall survival, as well as cancer-specific survival and greater risk of local recurrence; anastomotic leakage had no significant difference in terms of distant recurrence.

There is only one meta-analysis on a similar topic, which was done by Mirnezami et al. [31] and published in 2011. There are many dissimilar characteristics between the two studies. First, the prior study included patients with both colon and rectal cancer. Because the incidence of anastomotic leakage after resection of colon cancer is very low (2.4 %) compared to resections for rectal cancer [32], our study specifically only included those patients with rectal cancers. Furthermore, the prior study included patients with stage IV colorectal cancer and palliative operations which confound the analysis. These factors were very likely to affect the long-term outcomes of patients [30, 33, 34], which is the reason for exclusion of colon cancer, emergency operations, patients with stage IV disease, and palliative operations in the present study. Second, the prior study analyzed time-to-event data as dichotomous and expressed effect size as an odds ratio. Our meta-analysis used methods of survival analysis and expressed effect size as an HR, which is considered the most appropriate means of summarizing time-to-event data [35]. Lastly, the present meta-analysis included more recently published articles than the analysis conducted by Mirnezami et al.

Some factors may explain our findings of anastomotic leakage being associated with high local recurrence, poorer overall survival, and poorer cancer-specific survival. Colorectal cancer exfoliates cancer cells remaining in the lumen of the bowel and from the large intestinal mucosa, potentially seeding the local environment after resection [36]. Although the rectal stump was routinely washed out, free malignant cells can be found in the anastomosis of the anterior resection [37, 38]. When anastomotic leakage occurs, these cells may lead to extraluminal tumor implantation and pelvic recurrence. Moreover, anastomotic leakage causes postoperative peritoneal and pelvic infection, which may enhance proliferation, migration, and invasion capacities of cancer cells as shown in cancer cell lines in vitro [39]. Furthermore, some studies found that peritoneal infection increased serum interleukin-6 (IL-6), vascular endothelial growth factor (VEGF), and C-reactive protein (CRP) concentrations, which are associated with poor overall and cancer-specific survival [40–42]. Postoperative adjuvant chemotherapy for patients undergoing oncologic resections for rectal cancer has provided significantly positive effects on overall survival and distant metastasis [43], but anastomotic leakage may prevent or delay the receipt of adjuvant chemotherapy. This may also explain poorer survival in patients with anastomotic leakage [44].

Even though heterogeneity was present in our study, we detected its major source through sensitivity analysis. Quality appraisal is incomplete in most reviews of prognosis studies [14], but our meta-analysis incorporated adequate quality assessment and included studies that achieved a high median score. In addition, there was no publication bias, suggesting that our conclusions are not an artifact of unpublished studies. As more evidence becomes available, the test power to provide reliable estimates of risk increases.

There are a number of limitations of our study that must be considered. First, different definitions of anastomotic leakage have been applied throughout the studies included in this meta-analysis, regardless of a definition and grading of anastomotic leakage being proposed by the International Study Group of Rectal Cancer in 2010 [13]. Moreover, some included studies only contained patients with clinical anastomotic leakage, while others contained patients with clinical and radiologic anastomotic leakage. Second, though the relationship between anastomotic leakage and outcome measures was analyzed using multivariate the Cox proportional hazards model, each study had different confounder variables. These two concerns may have influenced the association of anastomotic leakage with recurrence or survival. Finally, although we concluded that anastomotic leakage did not increase distant recurrence, only three studies addressed this topic, and patient sample size was relatively small (*n* = 2397), indicating lesser statistical power.

In conclusion, based on available evidence, anastomotic leakage is associated with a greater risk of local recurrence and poorer overall and cancer-specific survival. These findings suggest that all attempts to decrease the incidence of anastomotic leakage should be employed when performing anterior resection of the rectum. Furthermore, close follow-up of patients with anastomotic leakage should be conducted. Whether local adjuvant radiation is beneficial in patients with anastomotic leakage is not known, but this special use of radiation therapy in this clinical situation might be a topic of interest in future studies.
